# Collisional positron acoustic soliton and double layer in an unmagnetized plasma having multi-species

**DOI:** 10.1038/s41598-022-10236-6

**Published:** 2022-04-19

**Authors:** Shahrina Akter, M. G. Hafez

**Affiliations:** 1 Department of Mathematics, Chittagong University of Engineering and Technology, Chattogram, 4349 Bangladesh; 2 Department of Mathematics, Premier University, Chattogram, Bangladesh

**Keywords:** Plasma physics, Space physics, Mathematics and computing

## Abstract

This paper explores the head-on collision between two-counter propagating positron acoustic solitons and double layers (DLs) in an unmagnetized collisionless plasma having mobile cold positrons fluid, immobile positive ions and ($r,\;q$)-distributed hot positrons, and hot electrons. By employing the extended Poincaré–Lighthill–Kuo method, the coupled Korteweg–de Vries (KdV), modified KdV (mKdV) and Gardner equations are derived to archive this goal. The effect of dimensionless parameters on the propagation characteristics of interacting KdV solitons (KdVSs), mKdV solitons (mKdVSs), Gardner solitons (GSs) and DLs are examined in detail by considering the limiting cases of ($r,\;q$)-distribution. It is noted that the interaction of GSs and DLs are reported for the first time. The outcomes might be comprehended and beneficial not only in space and astrophysical environments but also in laboratory studies.

## Introduction

The existence of electron–positron (EP) and electron–positron-ion (EPI) plasmas in space and astrophysical environments (SAEs) are well confirmed. Besides, EP pair production can be found not only just in SAEs but also in laboratories, where positrons can be employed to study particle movement in tokamark plasmas^1,2^. Producing space or astrophysical-like plasmas in the laboratory for reporting the basic features of such plasma is not an easy challenge for researchers. But, one may study such physical scenarios by proposing theoretical model equations (TMEs) under various types of plasma assumptions. One can then implement the exhausting mathematical procedures (EMPs) to solve the TMEs. Many researchers^3–22^ have already studied the features of various kinds of acoustic wave phenomena by assuming EPI plasma involving different distributed lighter plasma particles via EMPs. Since various plasma species are inhabited in the different regions of phase space^23^. It is feasible to incorporate varied temperatures of the species while designing quasi-stationary nonlinear structures in a multi-species plasma models. For instance, the basic features of electron acoustic propagation in an unmagnetized collisionless EPI plasma were studied in Ref.^24^ by taking $N_{ce0} \approx \left( {0.1 - 0.4} \right)\;{\text{cm}}^{ - 3}$, $N_{p0} \approx \left( {1.5 - 3} \right)\;{\text{cm}}^{ - 3}$, $N_{he0} \approx 1.53\;{\text{cm}}^{ - 3}$, $T_{he} \approx \left( {200 - 1000} \right)\;{\text{eV}}$, and $T_{p} \approx \left( {200 - 1000} \right)\;{\text{eV}}$ into account, where $N_{J0}$ and $T_{J}$ is the unperturbed density and temperature ($J = ce \left( {he} \right)$) and $p$ for cold (hot) electron and positron into account.

On the other hand, positron acoustic (PA) waves are one kind of electrostatic waves in which the inertia is controlled by the mobile cold positrons (MCPs) mass. The thermal pressure of other charged lighter particles (e.g. hot positrons (HPs) and hot electrons (HEs)) are contributed to the production of the restoring force in the plasmas. One can study the dynamics of positively charged inertial MCPs fluid based on the condition $V_{tMCP} \ll \omega {/}k \ll V_{tHP,HE}$ and their frequency much higher than frequency of ion ($V_{tj} , j = MCP, HP, HE$ are the thermal velocity for plasma species). However, various kinds of plasmas are produced by consisting of substantially high energy particles in most of SAEs. Such energetic particle mainly arises with the control of exterior forces by acting on the wave-particle interactions or natural space. PAMELA satellite has already confirmed that the prosperity of positrons in the cosmic radiation with energies $1.5 - 100 \;{\text{GeV}}$^25^. As a result, the propagation of PA waves having multi-species has been reported by many researchers^7–17^. For instance, Shah and Rakha^8^ examined the wave phenomena excitation by positron showers of astrophysical naturally doped superthermal plasmas; Shah et al.^9^ investigated the nonplenar PA shock wave excitations that observed in some object of SAEs; Saha et al.^7^ investigated the PAWs for understanding in the physics behind auroral acceleration regions; and Saha and Tamang^11^ studied the analysis of PA waves in multi-species plasmas for understanding the dynamic features of the cosmic rays. They have showed that the propagation of PA waves might be very beneficial to understand the nature of various SAEs, such as neutron starts^26^, active galactic nuclei^27^, pulsar magmetosphere^28^, solar wind, ionosphere, lower part of magnetosphere, and auroral acceleration regions^11^. Due to the existence of PA waves in many SAEs, Tribeche and his research group^14,15^ have reported only the electrostatic PA one sided propagating solitary waves and DLs in the four-component EPI plasmas by the mixture of immobile positive ions (IMPIs), MCPs, and isothermal HPs as well as HEs. Later, Mamun and his research group^12,21^ have reported the roles of superthermal as well as nonthermal electrons and positrons on PA solitons and DLs in an unmagnetized EPI plasma by deriving only the single Korteweg–de Vries (KdV), modified KdV (mKdV), and Gardner equations. In only a few works^13,17^, the collisional wave phenomena between two-counter propagating PA solitons have been examined in the aforementioned plasmas either considering isothermal or superthermal HEs and HPs. El-Shamy et al.^13^ have studied the characteristics of the head-on collision (HOC) between two PA solitary waves by deriving only the coupled KdV equations in a four component EPI plasma. Recently, Alam et al.^17^ have examined the HOC between two PA solitons described by only the coupled KdV and mKdV equations for the model equations proposed in Ref.^12^. Such equations are unable to describe the features of collisional wave phenomena in the plasmas when the nonlinear coefficients of these equations are equal to zero. It is provided that there are still now so many possibilities to report the unrevealed physical issues in such plasmas by deriving new coupled nonlinear evolution equations (NLEEs). However, the subsistence of flat top or shoulders cannot be ignored as model by either with the Maxwellian or kappa distribution as observed in many SAEs, e.g. the Earth’s magnetosphere and magnetosheath^29,30^, polar cusp^31^, etc. In such cases, one requires to use the more suitable generalized non-Maxwellian velocity distribution function^32,33^ for the lighter species. Such distribution function has two spectral indices in which one is performing as superthermality index on the tail of the velocity curve and another one is indicating the high energy particles on a broad shoulder of the velocity curve. It is therefore important to study the PA resonance wave phenomena described by new coupled NLEEs with the presence of the generalized non-Maxwellian velocity distributed lighter species.

Further, the most important striking properties of solitons are their asymptotic preservation of shape once they undergo a collision, as first described by Zabusky and Kruskal^34^. As a result, two different soliton interactions occur in not only one-dimensional but also the quasi-one-dimensional system in which one is the overtaking collision and the other is the head-on collision (HOC)^35^. The overtaking collision of solitary waves can be studied by formulating only the multi-wave solutions travel in the same direction of the single NLEEs (e.g. KdV equation, Burgers equation, etc.). In overtaking collision, the angle between the interacting waves is also zero. Very recently, Hafez et al.^36^ have reported overtaking collision of traveling wave by formulating the multi-solutions of the Burgers equation. They have clearly mentioned in their findings that the overtaking wave phenomena are propagating in the same direction. However, for a HOC between two-counter propagating waves, one must search for the development of waves traveling to both sides. Note that the basic features of small but finite amplitude wave phenomena are studied by the reductive perturbation approach^37^, whereas the Sagdeev potential approach^38^ is usually implemented to study the large amplitude wave phenomena. Both approaches are only provided the single NLEEs. By implementing these methods, it is not possible to study HOC between two waves propagating towards each other. Because, two oppositely propagating solitary wave can exceed through each other without suffering any interface apart from for time delayed in their positions. Besides, the HOC between two-solitons occur when the angle between two counter propagating solitons is π^35^. One can also consider the limiting case of long-wavelength approximation, like the reductive perturbation approach, by assuming the interaction between two solitons having small but finite amplitude is weak. As a result, one can expect the HOC to be quasi-elastic and there will support phase lags of both solitons after the collision. Hence, one needs to employ a suitable asymptotic expansion to solve the original model equations. In such situations, one can employ the asymptotic expansion approach, that is, the extended Poincare–Lighthill–Kuo (ePLK) method^39–41^ to study the feature of HOC wave phenomena from the considered plasma model equations.

Harvey et al.^42^ have reported experimentally and numerically the HOC between two-counter propagating solitons of equal amplitudes in strongly coupled plasma. They have found that the solitons are delayed after the collision and a longer delay is obtained for solitons with higher amplitude. They have also checked the accuracy of the experimental measurements by considering the KdV model^43^ and mentioned that the accuracy is not high enough to check whether the delay is proportional to the square root of the initial amplitude. In most of the previous studies^13,22,43–49^, researchers have studied the colliding same amplitude solitons and their phase shift by considering the square root of the initial amplitude, while the amplitude of solitons is strongly dependent on the plasma parameters. As a result, there are still now possibilities for studying the HOC wave phenomena between two solitons having proper amplitudes via the weakly nonlinear theories. Besides, Zhang et al.^50^ have clearly described the application scope of the ePLK method to study the HOC between two waves by deriving two-sided KdV equations via the Particle-in-cell numerical method. They have concluded that the ePLK method is only valid when the amplitudes of both the colliding solitary waves described by the coupled KdV equations are small enough. In Refs.^42,50^, researchers have also mentioned that one can study the basic features of colliding some small but finite amplitude secondary structures by taking higher order correction in the collision event. Note that the amplitudes of such colliding secondary structures are comparatively higher rather than colliding KdV solitons. Moreover, the coupled KdV and mKdV equations are not applicable to study the collisional wave phenomena properly not only around but also at the critical values for any specific plasma parameters. Also, such equations admit only collisional wave phenomena between two solitons but not double layers (DLs). In such a case, one can derive the coupled Gardner equations to study the collisional wave phenomena between two solitons and DLs propagating towards each other. To the best of our knowledge, no research work has been studied the collisional PA two-counter propagating solitons and DLs not only around the critical values but also at the critical values described by the coupled Gardner equations in any plasma environment. Thus, the work presented in this article explores the collisional PA solitons and DLs described by KdV, mKdV and Gardner equations in an unmagnetized plasma consisting of IMPIs, MCPs, and double spectral index non-Maxwellian velocity distributed HPs as well as HEs. The effect of plasma parameters on the collisional PA two-counter propagating KdV solitons (KdVSs), mKdV solitons (mKdVSs), Gardner solitons (GSs) and Gardner DLs (DLs) are described along with graphical representation.

## Model equations

An unmagnetized collisonless mult-species plasma system is considered by the mixture of MCPs with mass $m_{cp}$, IMPIs, HPs and HEs along with $N_{e0} = N_{cp0} + N_{hp0} + N_{i0}$, where $N_{i0} , N_{e0}$, $N_{cp0 }$ and $N_{hp0}$ are the unperturbed IMPIs, HEs, MCP and HPs number densities, respectively. It is noted that HPs and HEs are assumed to follow the generalized non-Maxwellian velocity distribution. Because the existence of flat top or shoulders would not be considered as model by either Maxwellian or kappa distribution as observed in some SAEs^29–33^. As a result, one can use the following double index non-Maxwellian velocity distribution^18,32,33^:

$$ f_{rq} \left( {V_{e} } \right) = \frac{a}{{\pi \left( {V_{te} } \right)^{3/2} }}\left[ {1 + \frac{1}{q - 1}\left( {\frac{{V_{e}^{2} - \frac{2e\phi }{{m_{e} }}}}{{b\left( {\frac{{2T_{e} }}{{m_{e} }}} \right)}}} \right)^{r + 1} } \right]^{ - q} , $$

where

$$ a = \frac{{3\Gamma \left( q \right) \times \left( {q - 1} \right)^{{ - \frac{3}{2 + 2r}}} }}{{4b^{3/2} \Gamma \left( {q - \frac{3}{2 + 2r}} \right) \times \Gamma \left( {1 + \frac{3}{2 + 2r}} \right)}} ,\;\;b = \frac{{3\left( {q - 1} \right)^{{ - \frac{3}{1 + r}}} \Gamma \left( {q - \frac{3}{2 + 2r}} \right) \times \Gamma \left( {\frac{3}{2 + 2r}} \right)}}{{2\Gamma \left( {q - \frac{3}{2 + 2r}} \right) \times \Gamma \left( {\frac{5}{2 + 2r}} \right)}}. $$

where $q$ and $r$ are the real parameters that measuring the superthermality on the tail of the velocity curve and the high energy particles on a broad shoulder of the velocity curve, respectively. It is noted that such parameters indicate the departure from Maxwellian and kappa equilibrium. One can easily recover the Maxwellian and kappa distribution by setting the limit $r = 0,\;q \to \infty$ and $r = 0, \;q \to \kappa + 1$, respectively. One can also use such distribution as the physically meaningful distributions by considering $q > 1$ and $q\left( {r + 1} \right) > 5/2$^18,33^. By integrating $f_{rq} \left( {V_{e} } \right)$ with the help of cylindrical coordinates, Ulla et al.^18^ have defined the total number HE density as

$$ N_{e} = N_{e0} \left[ {1 + \Pi_{1} \phi + \Pi_{2} \phi^{2} + \Pi_{3} \phi^{3} + \cdots } \right]. $$

where

$$ \begin{aligned} \Pi_{1} & = \frac{{\left( {q - 1} \right)^{{ - \frac{1}{1 + 
r}}} \Gamma \left( {q - \frac{1}{2 + 2r}} \right) \times \Gamma \left( {\frac{1}{2 + 2r}} \right)}}{{2b\Gamma \left( {\frac{3}{2 + 2r}} \right) \times \Gamma \left( {q - \frac{3}{2 + 2r}} \right)}} , \\ \Pi_{2} & = \frac{{ - \left( {q - 1} \right)^{{ - \frac{2}{1 + r}}} \Gamma \left( {q + \frac{1}{2 + 2r}} \right) \times \Gamma \left( {\frac{ - 1}{{2 + 2r}}} \right)}}{{8b^{2} \Gamma \left( {\frac{3}{2 + 2r}} \right) \times \Gamma \left( {q - \frac{3}{2 + 2r}} \right)}}, \\ \Pi_{3} & = \frac{{\left( {q - 1} \right)^{{ - \frac{3}{1 + r}}} \Gamma \left( {q + \frac{3}{2 + 2r}} \right) \times \Gamma \left( {\frac{ - 3}{{2 + 2r}}} \right)}}{{16b^{3} \Gamma \left( {\frac{3}{2 + 2r}} \right) \times \Gamma \left( {q - \frac{3}{2 + 2r}} \right)}}, \ldots , \\ \end{aligned} $$

where $\phi$ is the normalized electrostatic potential. Since positron is the opposite charge particle of electron, the total number HPs density can be written as $N_{hp} = N_{hp0} \left[ {1 - \Pi_{1} \phi + \Pi_{2} \phi^{2} - \Pi_{3} \phi^{3} + \cdots } \right].$ To study the collisional PA wave phenomena, the hydrodynamic fluid equations in the dimensionless forms are obtained as below.

1 $$ \frac{\partial }{\partial t}N_{cp} \left( {z,t} \right) + \frac{\partial }{\partial z}\left[ {N_{cp} \left( {z,t} \right)U_{cp} \left( {z,t} \right)} \right] = 0{ }, $$

2 $$ \frac{\partial }{\partial t}U_{cp} \left( {z,t} \right) + U_{cp} \left( {z,t} \right)\frac{\partial }{\partial z}U_{cp} \left( {z,t} \right) = - \frac{\partial }{\partial z} \phi \left( {z,t} \right), $$

3 $$ \begin{aligned} \frac{{\partial^{2} }}{{\partial z^{2} }}\phi \left( {z,t} \right) & = - N_{cp} - N_{hc} \left[ {1 - \Pi_{1} \delta \phi \left( {z,t} \right) + \Pi_{2} \left( {\delta \phi \left( {z,t} \right)} \right)^{2} - \Pi_{3} \left( {\delta \phi \left( {z,t} \right)} \right)^{3} } \right] \\ & \quad + N_{ec} \left[ {1 + \Pi_{1} \sigma \phi \left( {z,t} \right) + \Pi_{2} \left( {\sigma \phi \left( {z,t} \right)} \right)^{2} + \Pi_{3} \left( {\sigma \phi \left( {z,t} \right)} \right)^{3} } \right] - N_{ic} = - \rho , \\ \end{aligned} $$

where

$$ N_{hc} = \frac{{N_{hp0} }}{{N_{cp0} }},\;N_{ec} = \frac{{N_{e0} }}{{N_{cp0} }},\; N_{ic} = \frac{{N_{i0} }}{{N_{cp0} }},\; \delta = \frac{{T_{ef} }}{{T_{hp} }},\; \sigma = \frac{{T_{ef} }}{{T_{e} }},\; T_{ef} = \frac{{T_{e} T_{hp} }}{{N_{ec} T_{hp} + N_{hc} T_{e} }}. $$

To formulate the dimensionless equations as in Eqs. (1)–(3), the characteristic scale of used variables and parameters are considered as (1) the number density of MCPs ($N_{cp}$) is normalized by its equilibrium value $N_{cp0}$, (2) MCPs fluid speed normalized by $\sqrt {k_{B} T_{ef} /m_{cp} }$ ($k_{B}$ is the Boltzmann constant, $T_{ef}$ is the effective temperature and $T_{e} (T_{hp} )$ is the HEs (HPs) temperature, (3) the electrostatic potential ($\phi$) is normalized by $k_{B} T_{ef} /e$, (4) time ($t$) is normalized by $\omega_{pc}^{ - 1} = \sqrt {m_{cp} {/}4\pi N_{cp0} e^{2} }$ and $\left( v \right)$ length (z) is normalized by $\lambda_{Dm} = \sqrt {k_{B} T_{ef} {/}4\pi N_{cp0} e^{2} }$.

## Mathematical analysis

To examine the collisional wave phenomena between two-counter propagating soliton and their corresponding phase shift, one can use the following starching coordinates^39–41,49^:

4 $$ \left( {\begin{array}{*{20}c} \xi \\ \eta \\ \tau \\ \end{array} } \right) = \left( {\begin{array}{*{20}c} {\varepsilon \left( {z - \lambda_{p} t} \right) + \varepsilon^{2} P_{0} \left( {\xi ,\eta ,\tau } \right) + \cdots } \\ {\varepsilon \left( {z + \lambda_{p} t} \right) + \varepsilon^{2} Q_{0} \left( {\xi ,\eta ,\tau } \right) + \cdots } \\ {\varepsilon^{3} t } \\ \end{array} } \right), $$

where $\xi$ and $\eta$ are the trajectories of soliton moving headed for each other, and $\lambda_{p}$ is the phase speed of PAWs and $0 < \varepsilon < 1$. Also, the perturbed variables can expand based on the concept of ePLK method^3,13,17^ as

5 $$ \left( {\begin{array}{*{20}c} {N_{cp} } \\ {U_{cp} } \\ \phi \\ \rho \\ \end{array} } \right) = \left( {\begin{array}{*{20}c} 1 \\ 0 \\ 0 \\ 0 \\ \end{array} } \right) + \mathop \sum \limits_{i = 1}^{\infty } \varepsilon^{i} \left( {\begin{array}{*{20}c} {N_{cp}^{\left( i \right)} } \\ {U_{cp}^{\left( i \right)} } \\ {\phi^{\left( i \right)} } \\ {\rho^{i} } \\ \end{array} } \right), $$

Implementing Eqs. (4) and (5) into Eqs. (1)–(3), one can determine the order of $\varepsilon$, that is, $O(\varepsilon^{r} )$ equations with the choice of $r$ consequently. The $O(\varepsilon^{2} )$ equations can be written in the matrix form as

6 $$ \left( {\begin{array}{*{20}c} { - \lambda_{p} D_{1} N_{cp}^{\left( 1 \right)} + D_{2} U_{cp}^{\left( 1 \right)} } \\ { - \lambda_{p} D_{1} U_{cp}^{\left( 1 \right)} + D_{2} \phi^{\left( 1 \right)} } \\ {N_{cp}^{\left( 1 \right)} } \\ \end{array} } \right) = \left( {\begin{array}{*{20}c} 0 \\ 0 \\ {{\mathcal{H}}_{1} \phi^{\left( 1 \right)} } \\ \end{array} } \right), $$

where

$$ D_{1} = \frac{\partial }{\partial \xi } - \frac{\partial }{\partial \eta },\;D_{2} = \frac{\partial }{\partial \xi } + \frac{\partial }{\partial \eta },\;{\mathcal{H}}_{1} = \frac{{\left( {N_{ec} \sigma + N_{hc} \delta } \right)\left( {q - 1} \right)^{{ - \frac{1}{1 + r}}} \Gamma \left( {q - \frac{1}{2 + 2r}} \right) \times \Gamma \left( {\frac{1}{2 + 2r}} \right)}}{{2b\Gamma \left( {\frac{3}{2 + 2r}} \right) \times \Gamma \left( {q - \frac{3}{2 + 2r}} \right)}}. $$

From Eq. (6), one formulates

7 $$ - \left( {\lambda_{p}^{2} {\mathcal{H}}_{1} - 1} \right)\left( {\frac{{\partial^{2} \phi^{\left( 1 \right)} }}{{\partial \xi^{2} }} + \frac{{\partial^{2} \phi^{\left( 1 \right)} }}{{\partial \xi^{2} }}} \right) + 2\left( {\lambda_{p}^{2} {\mathcal{H}}_{1} + 1} \right)\frac{{\partial^{2} \phi^{\left( 1 \right)} }}{\partial \xi \partial \eta } = 0, $$

To solve Eq. (7), one considers $\lambda_{p}^{2} {\mathcal{H}}_{1} - 1 = 0$ and yields

8 $$ \lambda_{p} = \left\{ {\frac{{2b\Gamma \left( {\frac{3}{2 + 2r}} \right) \times \Gamma \left( {q - \frac{3}{2 + 2r}} \right)}}{{\left( {N_{ec} \sigma + N_{hc} \delta } \right) \times \left( {q - 1} \right)^{{ - \frac{1}{1 + r}}} \Gamma \left( {q - \frac{1}{2 + 2r}} \right) \times \Gamma \left( {\frac{1}{2 + 2r}} \right)}}} \right\}^{1/2} . $$

As a result, the solution of Eq. (6) is determined as

9 $$ \left( {\begin{array}{*{20}c} {\phi^{\left( 1 \right)} } \\ {N_{cp}^{\left( 1 \right)} } \\ {U_{cp}^{\left( 1 \right)} } \\ \end{array} } \right) = \left( {\begin{array}{*{20}c} {\phi_{l}^{\left( 1 \right)} \left( {\xi ,\tau } \right) + \phi_{r}^{\left( 1 \right)} \left( {\eta ,\tau } \right)} \\ {\frac{1}{{\lambda_{p}^{2} }}\left[ {\phi_{l}^{\left( 1 \right)} \left( {\xi ,\tau } \right) + \phi_{r}^{\left( 1 \right)} \left( {\eta ,\tau } \right)} \right]} \\ {\frac{1}{{\lambda_{p} }}\left[ {\phi_{l}^{\left( 1 \right)} \left( {\xi ,\tau } \right) - \phi_{r}^{\left( 1 \right)} \left( {\eta ,\tau } \right)} \right]} \\ \end{array} } \right). $$

The $O(\varepsilon^{3} )$ equations gives

10 $$ \left( {\begin{array}{*{20}c} { - \lambda_{p} D_{1} N_{cp}^{\left( 2 \right)} + D_{2} U_{cp}^{\left( 2 \right)} + D_{2} \left( {N_{cp}^{\left( 1 \right)} U_{cp}^{\left( 1 \right)} } \right)} \\ { - \lambda_{p} D_{1} U_{cp}^{\left( 2 \right)} + \frac{1}{2}D_{2} \left\{ {U_{cp}^{\left( 1 \right)} } \right\}^{2} + D_{2} \phi^{\left( 2 \right)} } \\ { - N_{cp}^{\left( 2 \right)} + {\mathcal{H}}_{1} \phi^{\left( 2 \right)} - {\mathcal{H}}_{2} \left\{ {\phi^{\left( 1 \right)} } \right\}^{2} } \\ \end{array} } \right) = \left( {\begin{array}{*{20}c} 0 \\ 0 \\ 0 \\ \end{array} } \right), $$

where

$$ {\mathcal{H}}_{2} = \frac{{ - \left( {q - 1} \right)^{{ - \frac{2}{1 + r}}} \Gamma \left( {q + \frac{1}{2 + 2r}} \right) \times \Gamma \left( {\frac{ - 1}{{2 + 2r}}} \right) \times \left( {N_{ec} \sigma^{2} - N_{hc} \delta^{2} } \right)}}{{8b^{2} \Gamma \left( {\frac{3}{2 + 2r}} \right) \times \Gamma \left( {q - \frac{3}{2 + 2r}} \right)}}. $$

Solving Eq. (10) along with the choice of $\phi_{l}^{\left( 2 \right)} \left( {\xi ,\tau } \right) \approx \phi_{l}^{\left( 2 \right)}$ and $\phi_{r}^{\left( 2 \right)} \left( {\eta ,\tau } \right) \approx \phi_{r}^{\left( 2 \right)}$, one determines

11 $$ \left( {\begin{array}{*{20}c} {\phi^{\left( 2 \right)} } \\ {N_{cp}^{\left( 2 \right)} } \\ {U_{cp}^{\left( 2 \right)} } \\ \end{array} } \right) = \left( {\begin{array}{*{20}c} {\phi_{l}^{\left( 2 \right)} + \phi_{r}^{\left( 2 \right)} } \\ {\frac{1}{{\lambda_{p}^{2} }}\left[ {\phi_{l}^{\left( 2 \right)} + \phi_{r}^{\left( 2 \right)} } \right] + \frac{3}{{2\lambda_{p}^{4} }}\left[ {\left\{ {\phi_{l}^{\left( 1 \right)} } \right\}^{2} + \left\{ {\phi_{r}^{\left( 1 \right)} } \right\}^{2} } \right]} \\ {\frac{1}{{\lambda_{p} }}\left[ {\phi_{l}^{\left( 2 \right)} - \phi_{r}^{\left( 2 \right)} } \right] + \frac{1}{{2\lambda_{p}^{3} }}\left[ {\left\{ {\phi_{l}^{\left( 1 \right)} } \right\}^{2} - \left\{ {\phi_{r}^{\left( 1 \right)} } \right\}^{2} } \right]} \\ \end{array} } \right). $$

and

12 $$ - \left( {\frac{3}{{2\lambda_{p}^{4} }} + {\mathcal{H}}_{1} } \right)\left\{ {\phi_{l}^{\left( 1 \right)} } \right\}^{2} = - \left( {\frac{3}{{2\lambda_{p}^{4} }} + {\mathcal{H}}_{1} } \right)\left\{ {\phi_{r}^{\left( 1 \right)} } \right\}^{2} = 0, $$

It is predicted from Eq. (12) that one can consider (1) $\phi_{l}^{\left( 1 \right)} = \phi_{r}^{\left( 1 \right)} = 0$ and (2) $\left( {\frac{3}{{2\lambda_{p}^{4} }} + {\mathcal{H}}_{1} } \right) = 0$.

### Coupled KdV equations and its stationary solutions

Based on the case (1), Eq. (11) reduces to

13 $$ \left( {\begin{array}{*{20}c} {\phi^{\left( 2 \right)} } \\ {N_{cp}^{\left( 2 \right)} } \\ {U_{cp}^{\left( 2 \right)} } \\ \end{array} } \right) = \left( {\begin{array}{*{20}c} {\phi_{l}^{\left( 2 \right)} + \phi_{r}^{\left( 2 \right)} } \\ {\frac{1}{{\lambda_{p}^{2} }}\left[ {\phi_{l}^{\left( 2 \right)} + \phi_{r}^{\left( 2 \right)} } \right]} \\ {\frac{1}{{\lambda_{p} }}\left[ {\phi_{l}^{\left( 2 \right)} - \phi_{r}^{\left( 2 \right)} } \right]} \\ \end{array} } \right). $$

For this case, $O(\varepsilon^{4} )$ equations yields

14 $$ \left( {\begin{array}{*{20}c} { - \lambda_{p} D_{1} N_{cp}^{\left( 3 \right)} + D_{2} U_{cp}^{\left( 3 \right)} } \\ { - \lambda_{p} D_{1} U_{cp}^{\left( 3 \right)} + D_{2} \phi^{\left( 3 \right)} } \\ {N_{cp}^{\left( 3 \right)} } \\ \end{array} } \right) = \left( {\begin{array}{*{20}c} 0 \\ 0 \\ {{\mathcal{H}}_{1} \phi^{\left( 3 \right)} } \\ \end{array} } \right), $$

It is noted that one may consider $N_{cp}^{\left( 3 \right)} = U_{cp}^{\left( 3 \right)} = \phi^{\left( 3 \right)} = 0$ because these perturb quantities are not involved in higher order equations. After exhausting calculations, the following coupled KdV equations are derived:

15 $$ \frac{{\partial \phi_{l}^{\left( 2 \right)} }}{\partial \tau } + A\phi_{l}^{\left( 2 \right)} \frac{{\partial \phi_{l}^{\left( 2 \right)} }}{\partial \xi } + B\frac{{\partial^{3} \phi_{l}^{\left( 2 \right)} }}{{\partial \xi^{3} }} = 0, $$

16 $$ \frac{{\partial \phi_{r}^{\left( 2 \right)} }}{\partial \tau } - A\phi_{r}^{\left( 2 \right)} \frac{{\partial \phi_{r}^{\left( 2 \right)} }}{\partial \eta } - B\frac{{\partial^{3} \phi_{r}^{\left( 2 \right)} }}{{\partial \eta^{3} }} = 0. $$

where

$$ A = \left[ {\frac{3}{{2\lambda_{p} }} - \frac{{\lambda_{p}^{3} \left( {q - 1} \right)^{{ - \frac{2}{1 + r}}} \Gamma \left( {q + \frac{1}{2 + 2r}} \right) \times \Gamma \left( {\frac{ - 1}{{2 + 2r}}} \right) \times \left( {N_{ec} \sigma^{2} - N_{hc} \delta^{2} } \right)}}{{8b^{2} \Gamma \left( {\frac{3}{2 + 2r}} \right) \times \Gamma \left( {q - \frac{3}{2 + 2r}} \right)}}} \right],\;B = \frac{{\lambda_{p}^{3} }}{2}. $$

When $r = 0$ and $q \to \kappa + 1$, the obtained nonlinear coefficient $\left( A \right)$ and dispersive coefficient $\left( B \right)$ of the coupled KdV equations are only equivalent to $A$ and $B$ of the earlier investigation in Ref.^17^. In addition, the phase functions are formulated as

17 $$ P_{0} = \frac{D}{C}\mathop \int \limits_{ - \infty }^{\eta } \phi_{l}^{\left( 2 \right)} \left( { \eta^{\prime},\tau } \right)d\eta^{\prime},\;\;Q_{0} = \frac{D}{C}\mathop \int \limits_{\infty }^{\xi } \phi_{r}^{\left( 2 \right)} \left( {\xi^{\prime},\tau } \right)d\xi^{\prime}, $$

where

$$ C = 2\lambda_{p} ,D = \left[ {\frac{1}{{2\lambda_{p} }} - \frac{{ - \lambda_{p}^{3} \left( {q - 1} \right)^{{ - \frac{2}{1 + r}}} \Gamma \left( {q + \frac{1}{2 + 2r}} \right) \times \Gamma \left( {\frac{ - 1}{{2 + 2r}}} \right) \times \left( {N_{ec} \sigma^{2} - N_{hc} \delta^{2} } \right)}}{{8b^{2} \Gamma \left( {\frac{3}{2 + 2r}} \right) \times \Gamma \left( {q - \frac{3}{2 + 2r}} \right)}}} \right]. $$

Now, the useful stationary wave solutions of Eqs. (15) and (16) are defined as

18 $$ \phi_{l}^{\left( 2 \right)} = \phi_{2a} {\text{sech}}^{2} \left\{ {\frac{{\left( {\xi - U_{0} \tau } \right)}}{{\phi_{2w} }}} \right\}, \;\;\phi_{r}^{\left( 2 \right)} = \phi_{2a} {\text{sech}}^{2} \left\{ {\frac{{\left( {\eta + U_{0} \tau } \right)}}{{\phi_{2w} }}} \right\}, $$

where $\phi_{2a} = \left( {3U_{0} {/}A} \right)$ and $\phi_{2w} = \sqrt {\left( {(4B{/}U_{0} } \right)}$ are the amplitudes and widths of the solitary waves traveling from each other from their initial positions and $U_{0}$ is the constant velocity of the reference frame. To apply the formulae $\nabla P_{0} = \varepsilon \left( {z - \lambda_{p} t} \right)\left. \right|_{\xi = 0, \eta \to - \infty } - \varepsilon \left( {z - \lambda_{p} t} \right)\left. \right|_{\xi = 0, \eta \to \infty }$ and $\nabla Q_{0} = \varepsilon \left( {z + \lambda_{p} t} \right)\left. \right|_{\xi = - \infty , \eta \to 0} - \varepsilon \left( {z + \lambda_{p} t} \right)\left. \right|_{\xi = \infty , \eta \to 0}$, one obtains

19 $$ \nabla P_{0} = - 2\varepsilon^{2} \frac{D}{C}\phi_{2a} \phi_{2w} , \;\;\nabla Q_{0} = 2\varepsilon^{2} \frac{D}{C}\phi_{2a} \phi_{2w} . $$

### Coupled mKdV equations and its stationary solutions

For case (2),

$$ \left( {\frac{3}{{2\lambda_{p}^{4} }} + {\mathcal{H}}_{2} } \right) = 0, $$

which yields the critical value (say $K_{v}$) of any specific parameter (say $N_{hc}$) as

20 $$ N_{hc} = K_{v} = - \frac{{3N_{ec} \delta \Pi_{1}^{2} + \Pi_{2} \sigma \pm \sqrt {6N_{ec} \sigma \delta \Pi_{1}^{2} \Pi_{2} + 6N_{ec} \delta \Pi_{1}^{2} \Pi_{2} + \sigma^{2} \Pi_{2}^{2} } }}{{3\Pi_{1}^{2} \sigma }}. $$

In order to examine the collisional wave phenomena between two soliton and phase shift around $K_{v}$, one may insert $\phi_{l}^{\left( 2 \right)} = \phi_{r}^{\left( 2 \right)} = 0$^49^. So, Eq. (11) is obtained as

21 $$ \left( {\begin{array}{*{20}c} {\phi^{\left( 2 \right)} } \\ {N_{cp}^{\left( 2 \right)} } \\ {U_{cp}^{\left( 2 \right)} } \\ \end{array} } \right) = \left( {\begin{array}{*{20}c} 0 \\ {\frac{3}{{2\lambda_{p}^{4} }}\left[ {\left\{ {\phi_{l}^{\left( 1 \right)} } \right\}^{2} + \left\{ {\phi_{r}^{\left( 1 \right)} } \right\}^{2} } \right]} \\ {\frac{1}{{2\lambda_{p}^{3} }}\left[ {\left\{ {\phi_{l}^{\left( 1 \right)} } \right\}^{2} - \left\{ {\phi_{r}^{\left( 1 \right)} } \right\}^{2} } \right]} \\ \end{array} } \right). $$

Based on the case (2), the $O\left( {\varepsilon^{3} } \right)$ equations are obtained as below.

22 $$ \begin{aligned} & \frac{{\partial N_{cp}^{\left( 1 \right)} }}{\partial \tau } - \lambda_{p} D_{1} N_{cp}^{\left( 3 \right)} + D_{2} U_{cp}^{\left( 3 \right)} + D_{2} \left[ {N_{cp}^{\left( 1 \right)} U_{cp}^{\left( 2 \right)} + N_{cp}^{\left( 2 \right)} U_{cp}^{\left( 1 \right)} } \right] - \lambda_{p} \left( {D_{1} P_{0} } \right)\frac{{\partial N_{cp}^{\left( 1 \right)} }}{\partial \xi } - \lambda_{p} \left( {D_{1} Q_{0} } \right)\frac{{\partial N_{cp}^{\left( 1 \right)} }}{\partial \eta } \\ & \quad + \left( {D_{2} P_{0} } \right)\frac{{\partial U_{cp}^{\left( 1 \right)} }}{\partial \xi } + \left( {D_{2} Q_{0} } \right)\frac{{\partial U_{cp}^{\left( 1 \right)} }}{\partial \eta } = 0, \\ \end{aligned} $$

23 $$ \begin{aligned} & \frac{{\partial U_{cp}^{\left( 1 \right)} }}{\partial \tau } - \lambda_{p} D_{1} U_{cp}^{\left( 3 \right)} + D_{2} \phi^{\left( 3 \right)} + D_{2} \left[ {U_{cp}^{\left( 1 \right)} U_{cp}^{\left( 2 \right)} } \right] - \lambda_{p} \left( {D_{1} P_{0} } \right)\frac{{\partial U_{cp}^{\left( 1 \right)} }}{\partial \xi } - \lambda_{p} \left( {D_{1} Q_{0} } \right)\frac{{\partial U_{cp}^{\left( 1 \right)} }}{\partial \eta } \\ & \quad + \left( {D_{2} P_{0} } \right)\frac{{\partial \phi^{\left( 1 \right)} }}{\partial \xi } + \left( {D_{2} Q_{0} } \right)\frac{{\partial \phi^{\left( 1 \right)} }}{\partial \eta } = 0, \\ \end{aligned} $$

24 $$ \left( {\frac{{\partial^{2} }}{{\partial \xi^{2} }} + \frac{{\partial^{2} }}{\partial \xi \partial \eta } + \frac{{\partial^{2} }}{{\partial \eta^{2} }}} \right)\phi^{\left( 1 \right)} = {\mathcal{H}}_{1} \phi^{\left( 3 \right)} - N^{\left( 3 \right)} + {\mathcal{H}}_{3} \left\{ {\phi^{\left( 1 \right)} } \right\}^{3} , $$

where

$$ {\mathcal{H}}_{3} = \frac{{\left( {q - 1} \right)^{{ - \frac{3}{1 + r}}} \Gamma \left( {q + \frac{3}{2 + 2r}} \right) \times \Gamma \left( {\frac{ - 3}{{2 + 2r}}} \right) \times \left( {N_{hc} \delta^{3} + N_{ec} \sigma^{3} } \right)}}{{16b^{3} \Gamma \left( {\frac{3}{2 + 2r}} \right) \times \Gamma \left( {q - \frac{3}{2 + 2r}} \right)}}. $$

Equations (22)–(24) can then be converted with the help of (21) to

25 $$ \left. {\begin{array}{*{20}r} \hfill { - \lambda_{p} D_{1} N_{cp}^{\left( 3 \right)} + D_{2} U_{cp}^{\left( 3 \right)} = S_{1} \left( {\phi_{l}^{\left( 1 \right)} ,\phi_{r}^{\left( 1 \right)} ,P_{0} ,Q_{0} } \right)} \\ \hfill { - \lambda_{p} D_{1} U_{cp}^{\left( 3 \right)} + \lambda_{p}^{2} D_{2} N_{cp}^{\left( 3 \right)} = S_{2} \left( {\phi_{l}^{\left( 1 \right)} ,\phi_{r}^{\left( 1 \right)} ,P_{0} ,Q_{0} } \right)} \\ \end{array} } \right\} $$

where

26 $$ \begin{aligned} S_{1} \left( {\phi_{l}^{\left( 1 \right)} ,\phi_{r}^{\left( 1 \right)} ,P_{0} ,Q_{0} } \right) & = \left[ { - \frac{1}{{\lambda_{p}^{2} }}\frac{{\partial \phi_{l}^{\left( 1 \right)} }}{\partial \tau } - \frac{6}{{\lambda_{p}^{5} }}\left\{ {\phi_{l}^{\left( 1 \right)} } \right\}^{2} \frac{{\partial \phi_{l}^{\left( 1 \right)} }}{\partial \xi }} \right] + \left[ { - \frac{1}{{\lambda_{p}^{2} }}\frac{{\partial \phi_{r}^{\left( 1 \right)} }}{\partial \tau } + \frac{6}{{\lambda_{p}^{5} }}\left\{ {\phi_{r}^{\left( 1 \right)} } \right\}^{2} \frac{{\partial \phi_{r}^{\left( 1 \right)} }}{\partial \eta }} \right] \\ & \quad + \left[ { - \frac{1}{{\lambda_{p}^{5} }}\left\{ {\phi_{r}^{\left( 1 \right)} } \right\}^{2} - \frac{2}{{\lambda_{p} }}\frac{{\partial P_{0} }}{\partial \eta }} \right]\frac{{\partial \phi_{l}^{\left( 1 \right)} }}{\partial \xi } + \left[ {\frac{1}{{\lambda_{p}^{5} }}\left\{ {\phi_{l}^{\left( 1 \right)} } \right\}^{2} + \frac{2}{{\lambda_{p} }}\frac{{\partial Q_{0} }}{\partial \xi }} \right]\frac{{\partial \phi_{r}^{\left( 1 \right)} }}{\partial \eta } + \frac{2}{{\lambda_{p}^{5} }}\phi_{l}^{\left( 1 \right)} \phi_{r}^{\left( 1 \right)} \left[ {\frac{{\partial \phi_{l}^{\left( 1 \right)} }}{\partial \xi } - \frac{{\partial \phi_{r}^{\left( 1 \right)} }}{\partial \eta }} \right], \\ \end{aligned} $$

27 $$ \begin{aligned} S_{2} \left( {\phi_{l}^{\left( 1 \right)} ,\phi_{r}^{\left( 1 \right)} ,P_{0} ,Q_{0} } \right) & = \left[ { - \frac{1}{{\lambda_{p} }}\frac{{\partial \phi_{l}^{\left( 1 \right)} }}{\partial \tau } - \left( {\frac{3}{{2\lambda_{p}^{4} }} - 3\lambda_{p}^{2} {\mathcal{H}}_{3} } \right)\left\{ {\phi_{l}^{\left( 1 \right)} } \right\}^{2} \frac{{\partial \phi_{l}^{\left( 1 \right)} }}{\partial \xi } - \lambda_{p}^{2} \frac{{\partial^{3} \phi_{l}^{\left( 1 \right)} }}{{\partial \xi^{3} }}} \right] \\ & \quad + \left[ {\frac{1}{{\lambda_{p} }}\frac{{\partial \phi_{r}^{\left( 1 \right)} }}{\partial \tau } - \left( {\frac{3}{{2\lambda_{p}^{4} }} - 3\lambda_{p}^{2} {\mathcal{H}}_{3} } \right)\left\{ {\phi_{r}^{\left( 1 \right)} } \right\}^{2} \frac{{\partial \phi_{r}^{\left( 1 \right)} }}{\partial \eta } - \lambda_{p}^{2} \frac{{\partial^{3} \phi_{r}^{\left( 1 \right)} }}{{\partial \eta^{3} }}} \right] + \left[ {\left( {\frac{1}{{2\lambda_{p}^{4} }} + 3\lambda_{p}^{2} {\mathcal{H}}_{3} } \right)\left\{ {\phi_{r}^{\left( 1 \right)} } \right\}^{2} - 2\frac{{\partial P_{0} }}{\partial \eta }} \right]\frac{{\partial \phi_{l}^{\left( 1 \right)} }}{\partial \xi } \\ & \quad + \left[ {\left( {\frac{1}{{2\lambda_{p}^{4} }} + 3\lambda_{p}^{2} {\mathcal{H}}_{3} } \right)\left\{ {\phi_{l}^{\left( 1 \right)} } \right\}^{2} - 2\frac{{\partial Q_{0} }}{\partial \xi }} \right]\frac{{\partial \phi_{r}^{\left( 1 \right)} }}{\partial \eta } + \left( {\frac{1}{{\lambda_{p}^{4} }} + 3\lambda_{p}^{2} {\mathcal{H}}_{3} } \right)\phi_{l}^{\left( 1 \right)} \phi_{r}^{\left( 1 \right)} \left[ {\frac{{\partial \phi_{l}^{\left( 1 \right)} }}{\partial \xi } + \frac{{\partial \phi_{r}^{\left( 1 \right)} }}{\partial \eta }} \right]. \\ \end{aligned} $$

The solution of the corresponding homogeneous equation of Eq. (25) provides the similar results to $O\left( {\varepsilon^{2} } \right)$ equations. Now, the particular integral of Eq. (25) can be defined as

28 $$ \left. \begin{aligned}    N_{{cp}}^{{\left( 3 \right)}}  & = \frac{1}{{4\lambda _{p}^{2} }}\left[ {\int\limits^{\eta } {\left( {\lambda _{p} S_{1}  + S_{2} } \right)d\eta ^{\prime } }  + \int\limits^{\xi } {\left( { - \lambda _{p} S_{1}  + S_{2} } \right)d\xi ^{\prime } } } \right]  \\    U_{{cp}}^{{\left( 3 \right)}}  & = \frac{1}{{4\lambda _{p} }}\left[ {\int\limits^{\eta } {\left( {\lambda _{p} S_{1}  + S_{2} } \right)d\eta ^{\prime } }  - \int\limits^{\xi } {\left( { - \lambda _{p} S_{1}  + S_{2} } \right)d\xi ^{\prime } } } \right]  \\   \end{aligned}  \right\}. $$

From Eqs. (26) and (27), one obtains

29 $$ \left. {\begin{array}{*{20}c} {\lambda_{p} S_{1} + S_{2} = f_{1} \left( \xi \right) + f_{2} \left( \eta \right) + f_{3} \left( \eta \right)\frac{{\partial \phi_{l}^{\left( 1 \right)} }}{\partial \xi } + f_{4} \left( \xi \right)\frac{{\partial \phi_{r}^{\left( 1 \right)} }}{\partial \eta } + \cdots } \\ { - \lambda_{p} S_{1} + S_{2} = g_{1} \left( \xi \right) + g_{2} \left( \eta \right) + g_{3} \left( \eta \right)\frac{{\partial \phi_{l}^{\left( 1 \right)} }}{\partial \xi } + g_{4} \left( \xi \right)\frac{{\partial \phi_{r}^{\left( 1 \right)} }}{\partial \eta } + \cdots } \\ \end{array} } \right\}, $$

where

$$ \begin{aligned} f_{1} \left( \xi \right) & = - \frac{2}{{\lambda_{p} }}\frac{{\partial \phi_{l}^{\left( 1 \right)} }}{\partial \tau } - \left( {\frac{15}{{2\lambda_{p}^{4} }} - 3\lambda_{p}^{2} {\mathcal{H}}_{3} } \right)\left\{ {\phi_{l}^{\left( 1 \right)} } \right\}^{2} \frac{{\partial \phi_{l}^{\left( 1 \right)} }}{\partial \xi } - \lambda_{p}^{2} \frac{{\partial^{3} \phi_{l}^{\left( 1 \right)} }}{{\partial \xi^{3} }}, \\ f_{2} \left( \eta \right) & = \frac{\partial }{\partial \eta }\left[ {\left( {\frac{3}{{2\lambda_{p}^{4} }} - \lambda_{p}^{2} {\mathcal{H}}_{3} } \right)\left\{ {\phi_{r}^{\left( 1 \right)} } \right\}^{3} - \lambda_{p}^{2} \frac{{\partial^{2} \phi_{r}^{\left( 1 \right)} }}{{\partial \eta^{2} }}} \right], \\ f_{3} \left( \eta \right) & = \left[ {\left( { - \frac{1}{{\lambda_{p}^{4} }} + 3\lambda_{p}^{2} {\mathcal{H}}_{3} } \right)\left\{ {\phi_{r}^{\left( 1 \right)} } \right\}^{2} - 4\frac{{\partial P_{0} }}{\partial \eta }} \right],\;\;f_{4} \left( \eta \right) = \left[ {\left( {\frac{1}{{3\lambda_{p}^{4} }} + 3\lambda_{p}^{2} {\mathcal{H}}_{3} } \right)\left\{ {\phi_{l}^{\left( 1 \right)} } \right\}^{2} } \right], \\ g_{1} \left( \xi \right) & = \frac{\partial }{\partial \xi }\left[ {\left( {\frac{3}{{2\lambda_{p}^{4} }} - \lambda_{p}^{2} {\mathcal{H}}_{3} } \right)\left\{ {\phi_{l}^{\left( 1 \right)} } \right\}^{3} - \lambda_{p}^{2} \frac{{\partial^{2} \phi_{l}^{\left( 1 \right)} }}{{\partial \xi^{2} }}} \right], \\ g_{2} \left( \eta \right) & = \frac{2}{{\lambda_{p} }}\frac{{\partial \phi_{r}^{\left( 1 \right)} }}{\partial \tau } - \left( {\frac{3}{{2\lambda_{p}^{4} }} - 3\lambda_{p}^{2} {\mathcal{H}}_{3} } \right)\left\{ {\phi_{r}^{\left( 1 \right)} } \right\}^{2} \frac{{\partial \phi_{r}^{\left( 1 \right)} }}{\partial \eta } - \lambda_{p}^{2} \frac{{\partial^{3} \phi_{r}^{\left( 1 \right)} }}{{\partial \eta^{3} }}, \\ g_{3} \left( \eta \right) & = \left[ {\left( {\frac{1}{{3\lambda_{p}^{4} }} + 3\lambda_{p}^{2} {\mathcal{H}}_{3} } \right)\left\{ {\phi_{r}^{\left( 1 \right)} } \right\}^{2} } \right],\;\;g_{4} \left( \xi \right) = \left[ {\left( { - \frac{1}{{\lambda_{p}^{4} }} + 3\lambda_{p}^{2} {\mathcal{H}}_{3} } \right)\left\{ {\phi_{l}^{\left( 1 \right)} } \right\}^{2} - 4\frac{{\partial Q_{0} }}{\partial \xi }} \right]. \\ \end{aligned} $$

To extract the secularity in $N_{cp}^{\left( 3 \right)}$ and $U_{cp}^{\left( 3 \right)}$, one must be considered $f_{1} \left( \xi \right) = 0$ and $g_{2} \left( \eta \right) = 0$. As a result, one obtains

30 $$ \left. {\begin{array}{*{20}c} {\frac{{\partial \phi_{l}^{\left( 1 \right)} }}{\partial \tau } + M_{1} M_{2} \left\{ {\phi_{l}^{\left( 1 \right)} } \right\}^{2} \frac{{\partial \phi_{l}^{\left( 1 \right)} }}{\partial \xi } + M_{2} \frac{{\partial^{3} \phi_{l}^{\left( 1 \right)} }}{{\partial \xi^{3} }} = 0} \\ {\frac{{\partial \phi_{r}^{\left( 1 \right)} }}{\partial \tau } - M_{1} M_{2} \left\{ {\phi_{r}^{\left( 1 \right)} } \right\}^{2} \frac{{\partial \phi_{r}^{\left( 1 \right)} }}{\partial \eta } - M_{2} \frac{{\partial^{3} \phi_{r}^{\left( 1 \right)} }}{{\partial \eta^{3} }} = 0.} \\ \end{array} } \right\}, $$

where

$$ M_{1} = \frac{15}{{2\lambda_{p}^{6} }} - 3{\mathcal{H}}_{3} , \;M_{2} = \frac{{\lambda_{p}^{3} }}{2}. $$

Equation (30) is known as the coupled mKdV equations. It is noted that the obtained coefficients $M_{1}$ and $M_{2}$ are in good agreement with the coefficient $\alpha$ and $B$ of the coupled mKdV equations of Ref.^17^ for only $r = 0$ and $q \to \kappa + 1$. The stationary solutions of these equations are given as

31 $$ \phi_{l}^{\left( 1 \right)} = \phi_{1a} {\text{sech}} \left\{ {\frac{{\left( {\xi - U_{0} \tau } \right)}}{{\phi_{1w} }}} \right\},\;\;\phi_{r}^{\left( 1 \right)} = \phi_{1a} {\text{sech}} \left\{ {\frac{{\left( {\eta + U_{0} \tau } \right)}}{{\phi_{1w} }}} \right\}, $$

where $\phi_{1a} = \left( {\sqrt {6U_{0} {/}M_{1} M_{2} } } \right)$ and $\phi_{1w} =  \sqrt {\left( {M_{2} {/}U_{0}} \right)}$ are the amplitudes and widths of the soliton traveling toward each other from their initial positions.

Now, Eq. (28) gives us

32 $$ \begin{aligned} N_{cp}^{\left( 3 \right)} & = \frac{1}{{4\lambda_{p}^{2} }}\left[ {\left( {\frac{3}{{2\lambda_{p}^{4} }} - \lambda_{p}^{2} {\mathcal{H}}_{3} } \right)\left( {\left\{ {\phi_{r}^{\left( 1 \right)} } \right\}^{3} + \left\{ {\phi_{l}^{\left( 1 \right)} } \right\}^{3} } \right) - \lambda_{p}^{2} \left( {\frac{{\partial^{2} \phi_{r}^{\left( 1 \right)} }}{{\partial \eta^{2} }} + \frac{{\partial^{2} \phi_{l}^{\left( 1 \right)} }}{{\partial \xi^{2} }}} \right)} \right] + \frac{1}{{4\lambda_{p}^{2} }}h_{1} \left( \eta \right)\frac{{\partial \phi_{l}^{\left( 1 \right)} }}{\partial \xi } \\ & \quad + \frac{1}{{4\lambda_{p}^{2} }}h_{2} \left( \xi \right)\frac{{\partial \phi_{r}^{\left( 1 \right)} }}{\partial \eta } + \frac{1}{{4\lambda_{p}^{2} }}\left( {\frac{1}{{3\lambda_{p}^{4} }} + 3\lambda_{p}^{2} {\mathcal{H}}_{3} } \right)\left[ {\phi_{r}^{\left( 1 \right)} \left\{ {\phi_{l}^{\left( 1 \right)} } \right\}^{2} + \phi_{l}^{\left( 1 \right)} \left\{ {\phi_{r}^{\left( 1 \right)} } \right\}^{2} } \right], \\ U_{cp}^{\left( 3 \right)} & = \frac{1}{{4\lambda_{p} }}\left[ {\left( {\frac{3}{{2\lambda_{p}^{4} }} - \lambda_{p}^{2} {\mathcal{H}}_{3} } \right)\left( {\left\{ {\phi_{r}^{\left( 1 \right)} } \right\}^{3} - \left\{ {\phi_{l}^{\left( 1 \right)} } \right\}^{3} } \right) - \lambda_{p}^{2} \left( {\frac{{\partial^{2} \phi_{r}^{\left( 1 \right)} }}{{\partial \eta^{2} }} - \frac{{\partial^{2} \phi_{l}^{\left( 1 \right)} }}{{\partial \xi^{2} }}} \right)} \right] + \frac{1}{{4\lambda_{p} }}h_{1} \left( \eta \right)\frac{{\partial \phi_{l}^{\left( 1 \right)} }}{\partial \xi } \\ & \quad - \frac{1}{{4\lambda_{p} }}h_{2} \left( \xi \right)\frac{{\partial \phi_{r}^{\left( 1 \right)} }}{\partial \eta } + \frac{1}{{4\lambda_{p} }}\left( {\frac{1}{{3\lambda_{p}^{4} }} + 3\lambda_{p}^{2} {\mathcal{H}}_{3} } \right)\left[ {\phi_{r}^{\left( 1 \right)} \left\{ {\phi_{l}^{\left( 1 \right)} } \right\}^{2} - \phi_{l}^{\left( 1 \right)} \left\{ {\phi_{r}^{\left( 1 \right)} } \right\}^{2} } \right], \\ \end{aligned} $$

where

$$ h_{1} = \left( { - \frac{1}{{\lambda_{p}^{4} }} + 3\lambda_{p}^{2} {\mathcal{H}}_{3} } \right)\mathop \int \limits^{\eta } \left\{ {\phi_{r}^{\left( 1 \right)} } \right\}^{2} d\eta^{\prime} - 4P_{0} ,\;h_{2} = \left( { - \frac{1}{{\lambda_{p}^{4} }} + 3\lambda_{p}^{2} {\mathcal{H}}_{3} } \right)\mathop \int \limits^{\xi } \left\{ {\phi_{l}^{\left( 1 \right)} } \right\}^{2} d\xi^{\prime} - 4Q_{0} . $$

It is confirmed that $h_{1} \left( \eta \right)$ and $h_{2} \left( \xi \right)$ must become secular in the next higher order equations and provides

33 $$ P_{0} = \frac{1}{4}\left( { - \frac{1}{{\lambda_{p}^{4} }} + 3\lambda_{p}^{2} {\mathcal{H}}_{3} } \right)\mathop \int \limits^{\eta } \left\{ {\phi_{r}^{\left( 1 \right)} } \right\}^{2} d\eta^{\prime},\;\;Q_{0} = \frac{1}{4}\left( { - \frac{1}{{\lambda_{p}^{4} }} + 3\lambda_{p}^{2} {\mathcal{H}}_{3} } \right)\mathop \int \limits^{\xi } \left\{ {\phi_{l}^{\left( 1 \right)} } \right\}^{2} d\xi^{\prime}. $$

Finally, one can also obtain the phase shifts due to the head-on collision between two mKdVSs as

34 $$ \nabla P_{0} = - \varepsilon^{2} \frac{1}{2}\left( { - \frac{1}{{\lambda_{p}^{4} }} + 3\lambda_{p}^{2} {\mathcal{H}}_{3} } \right)\phi_{1a} \phi_{1w} , \;\;\nabla Q_{0} = \varepsilon^{2} \frac{1}{2}\left( { - \frac{1}{{\lambda_{p}^{4} }} + 3\lambda_{p}^{2} {\mathcal{H}}_{3} } \right)\phi_{1a} \phi_{1w} . $$

### Coupled Gardner equations and its stationary solutions

It is noted that Eq. (30) is not helpful to study the resonance wave phenomena at $K_{v}$. At this point, one considers

$$ A \sim A_{0} = S\left( {\frac{\partial A}{{\partial N_{hc} }}} \right)_{{N_{hc} = K_{v} }} \left| {N_{hc} - K_{v} } \right| = \varepsilon SD, $$

where $\left| {N_{hc} - K_{v} } \right| \sim \varepsilon$ because it’s a very small quantity and $S \sim 1\left( { - 1} \right)$ for $N_{hc} > K_{v}$ ($N_{hc} < K_{v}$). Hence, one can re-determine the $O\left( {\varepsilon^{3} } \right)$ equation from Eq. (3) by adding $\rho_{3} \sim - \varepsilon^{3} \frac{1}{2}SD\left\{ \phi \right\}^{2}$ and yields

35 $$ \left( {\frac{{\partial^{2} }}{{\partial \xi^{2} }} + \frac{{\partial^{2} }}{\partial \xi \partial \eta } + \frac{{\partial^{2} }}{{\partial \eta^{2} }}} \right)\phi^{\left( 1 \right)} = {\mathcal{H}}_{1} \phi^{\left( 3 \right)} - N^{\left( 3 \right)} + \frac{SD}{2}\left\{ {\phi^{\left( 1 \right)} } \right\}^{2} + {\mathcal{H}}_{3} \left\{ {\phi^{\left( 1 \right)} } \right\}^{3} . $$

Simplifying Eqs. (22), (23) and (35) in the similar procedure that are given in Eqs. (25)–(35), one can easily derive the following coupled Gardner equations:

36 $$ \left. {\begin{array}{*{20}c} {\frac{{\partial \phi_{l}^{\left( 1 \right)} }}{\partial \tau } + SDM_{2} \phi_{l}^{\left( 1 \right)} \frac{{\partial \phi_{l}^{\left( 1 \right)} }}{\partial \xi } + M_{1} M_{2} \left\{ {\phi_{l}^{\left( 1 \right)} } \right\}^{2} \frac{{\partial \phi_{l}^{\left( 1 \right)} }}{\partial \xi } + M_{2} \frac{{\partial^{3} \phi_{l}^{\left( 1 \right)} }}{{\partial \xi^{3} }} = 0} \\ {\frac{{\partial \phi_{r}^{\left( 1 \right)} }}{\partial \tau } - SDM_{2} \phi_{r}^{\left( 1 \right)} \frac{{\partial \phi_{r}^{\left( 1 \right)} }}{\partial \xi } - M_{1} M_{2} \left\{ {\phi_{r}^{\left( 1 \right)} } \right\}^{2} \frac{{\partial \phi_{r}^{\left( 1 \right)} }}{\partial \eta } - M_{2} \frac{{\partial^{3} \phi_{r}^{\left( 1 \right)} }}{{\partial \eta^{3} }} = 0. } \\ \end{array} } \right\}. $$

The soliton solutions of Eq. (36) can be written as

37 $$ \left. {\begin{array}{*{20}c} {\phi_{l}^{\left( 1 \right)} = \left\{ {\frac{1}{{\phi_{a1} }} - \left( {\frac{1}{{\phi_{a2} }} - \frac{1}{{\phi_{a1} }}} \right)\cosh^{2} \frac{{\left( {\xi - U_{0} \tau } \right)}}{{\phi_{gw} }}} \right\}^{ - 1} } \\ {\phi_{r}^{\left( 1 \right)} = \left\{ {\frac{1}{{\phi_{a1} }} - \left( {\frac{1}{{\phi_{a2} }} - \frac{1}{{\phi_{a1} }}} \right)\cosh^{2} \left( {\frac{{\eta + U_{0} \tau }}{{\phi_{gw} }}} \right)} \right\}^{ - 1} } \\ \end{array} } \right\}, $$

where $$\phi_{a1,a2} = - \left( {SD{/}M_{1} } \right) \left[ {1 \mp \sqrt {1 + U_{0} {/}\left( {S^{2} D{/}6M_{1} } \right) } } \right]$$, $$U_{0} = \left( {SDM_{2} } \right)\phi_{a1,a2} + \left( {M_{1} M_{2} {/}6} \right)$$ and $$\phi_{gw} = 2{/}\sqrt { - M_{1} \phi_{a1,a2} {/}6 }$$.

However, the DL solution of Eq. (36) is defined as

38 $$ \phi_{l}^{\left( 1 \right)} = \frac{{\phi_{gD} }}{2}\left\{ {1 + \tanh \left( {\frac{{\xi - U_{0} \tau }}{{\phi_{Dw} }}} \right)} \right\},\;\;\phi_{r}^{\left( 1 \right)} = \frac{{\phi_{gD} }}{2}\left\{ {1 - \tanh \left( {\frac{{\eta + U_{0} \tau }}{{\phi_{Dw} }}} \right)} \right\}, $$

where $\phi_{gD} = \left( {6U_{0} {/}SM_{2} } \right) \left( {U_{0} = - S^{2} DM_{2} {/}6M_{1} } \right)$ is the height and $\phi_{DW} = 2{/}\phi_{gD} \sqrt { - M_{1} {/}6 }$ is thickness of the DL. Also, the phase functions can be obtained by the following:

39 $$ \left. \begin{aligned}    P_{0}  & = \frac{1}{4}\left( { - \frac{1}{{\lambda _{p}^{4} }} + 3\lambda _{p}^{2} {\mathcal{H}}_{3} } \right)\int\limits_{{}}^{\eta } {\left\{ {\phi _{r}^{{\left( 1 \right)}} \left( {\eta ^{\prime } ,\tau } \right)} \right\}^{2} d\eta ^{\prime } }  - \frac{1}{4}\lambda _{p}^{2} SD\int\limits_{{}}^{\eta } {\phi _{r}^{{\left( 1 \right)}} \left( {\eta ^{\prime } ,\tau } \right)d\eta ^{\prime } }  \\     Q_{0} & = \frac{1}{4}\left( { - \frac{1}{{\lambda _{p}^{4} }} + 3\lambda _{p}^{2} {\mathcal{H}}_{3} } \right)\int\limits_{{}}^{\xi } {\left\{ {\phi _{l}^{{\left( 1 \right)}} \left( {\xi ^{\prime } ,\tau } \right)} \right\}^{2} d\xi ^{\prime } }  - \frac{1}{4}\lambda _{p}^{2} SD\int\limits_{{}}^{\xi } {\left\{ {\phi _{l}^{{\left( 1 \right)}} \left( {\xi ^{\prime } ,\tau } \right)} \right\}d\xi ^{\prime } }  \\   \end{aligned}  \right\}. $$

## Results and discussion

To study the interaction of two-counter propagating PA not only KdVSs and mKdVSs but also GSs and DLs in the proposed plasmas, the nonlinear coupled KdV, mKdV, and Gardner equations have been derived by employing the ePLK method. Note that the coupled Gardner equations are derived for the first time. Based on the useful stationary wave solutions of these equations, the effects of $N_{hc}$ $(N_{hp0} {/}N_{cp0} )$, $N_{ec}$ ($N_{e0} {/}N_{cp0}$), $N_{ic}$ ($N_{i0} {/}N_{cp0}$), $\delta$ ($T_{ef} {/}T_{hp}$), ${ }\sigma$ ($T_{ef} {/}T_{e}$), $r$ and $q$ on the collisional PA solitons and DLs are described and presented graphically. In this investigation, the parametric values of these parameters are considered based on the Refs.^51–54^ those are reliable not only in SAEs but also in laboratory plasmas.

**Figure 1:**
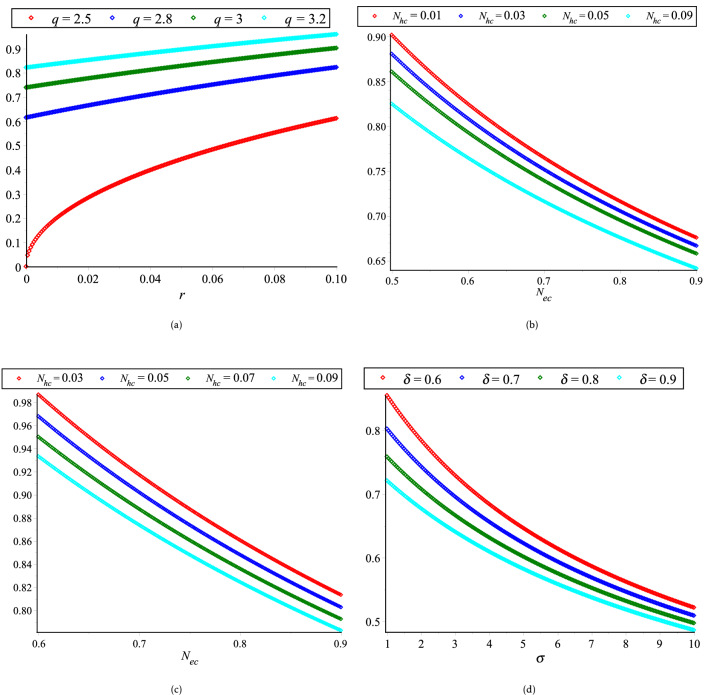
Variation of *λ*_*p*_ with regards to (**a**) *r* for different values of *q* with *N*_*hc*_ = 0.09, *N*_*ec*_ = 0.65, *δ* = 0.8 and *σ* = 1; (**b**) *N*_*ec*_ for different values of *N*_*hc*_ with *r* = 0, *q* = 3, *δ* = 0.8 and *σ* = 1; (**c**) *N*_*ec*_ for different values of *N*_*hc*_ with *r* = 0.1, *q* = 3, *δ* = 0.8 and *σ* = 1; and (**d**) *σ* for different values of *δ* with *N*_*hc*_ = 0.09, *N*_*ec*_ = 0.65, *q* = 3 and *r* = 0.01.

The effect of $N_{hc}$, $N_{ec}$, $\delta$, ${ }\sigma$, $r$ and $q$ on the phase velocity ($\lambda_{p}$) are displayed in Fig. 1a–d. The phase velocity of PA waves is increased (decreased) with the increase of $q$ and $r$ ($N_{hc}$, $N_{ec}$, $\delta$ and $\sigma$). It is identified that the phase velocity is strongly dominated by the superthermality on the tail of the velocity curve and the high energy particles on a broad shoulder of the velocity rather than the density and temperature ratios. It is also found that $\lambda_{p}$ is increased exponentially (red curve of Fig. 1a) with the increase of superthermal parameter of HEs and HPs ($r = 0$), which is in good agreement with the Ref.^12^.

**Figure 2:**
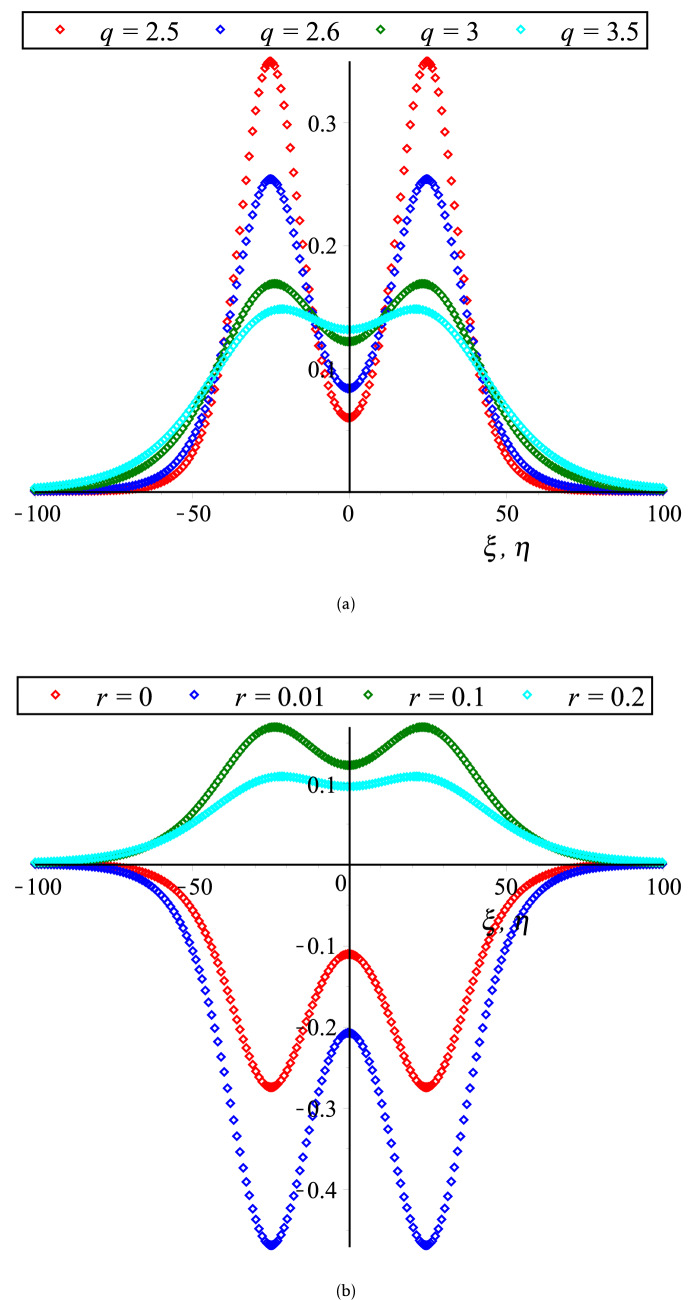
Collisional PA KdVSs $\left[ {\phi^{{({1})}} = \phi_{l}^{{({1})}} + \phi_{r}^{{({1})}} } \right]$ for different values of (**a**) *q* (*r* = 0.1) and (**b**) *r* (*q* = 3). The remaining dimensionless parameters are chosen as *N*_*hc*_ = 0.0015, *N*_*ec*_ = 0.5, *σ* = 0.5 and *δ* = 1 with *U*_0_ = 0.01 and *τ* = 2500.

**Figure 3:**
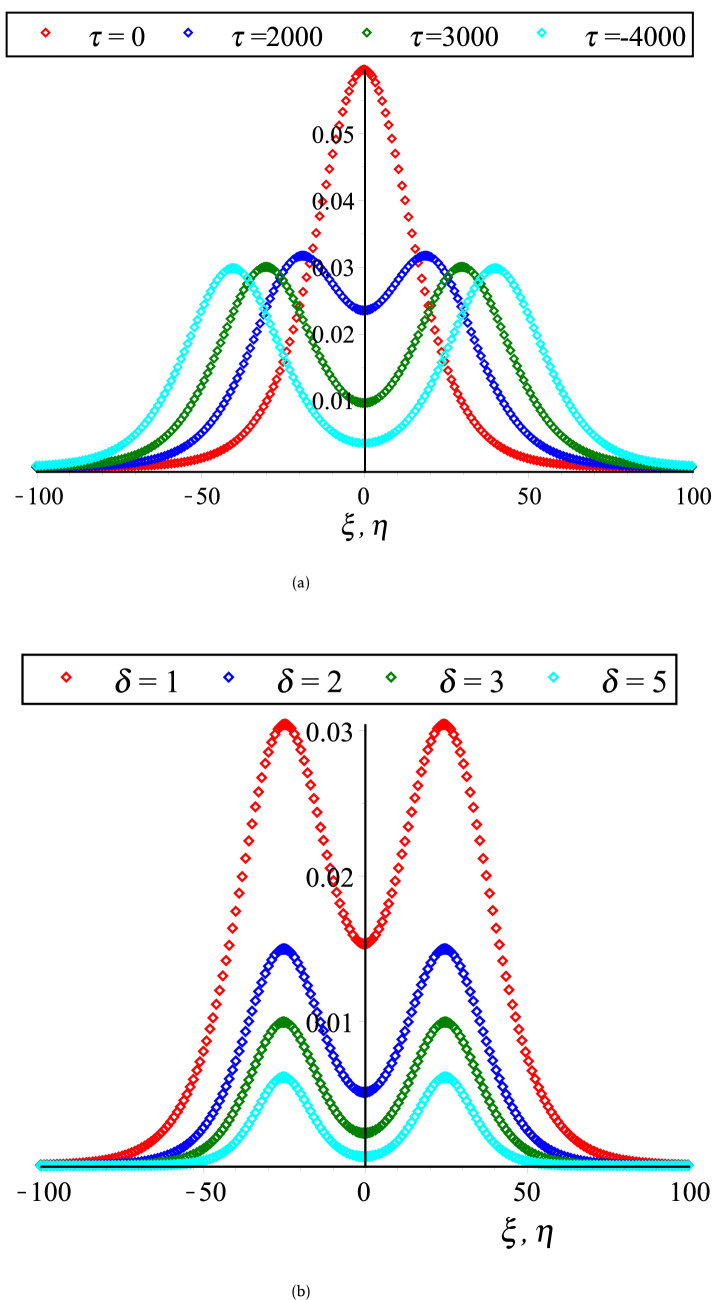
Collisional PA KdVSs $\left[ {\phi^{{({1})}} = \phi_{l}^{{({1})}} + \phi_{r}^{{({1})}} } \right]$ for different values of (**a**) *τ* (*δ* = 1) and (**b**) *δ* (*τ* = 2500). The remaining dimensionless parameters are chosen as *N*_*hc*_ = 0.08, *N*_*ec*_ = 0.5 and *σ* = 0.5 with *U*_0_ = 0.01, *r* = 0.1 and *q* = 3.

**Figure 4:**
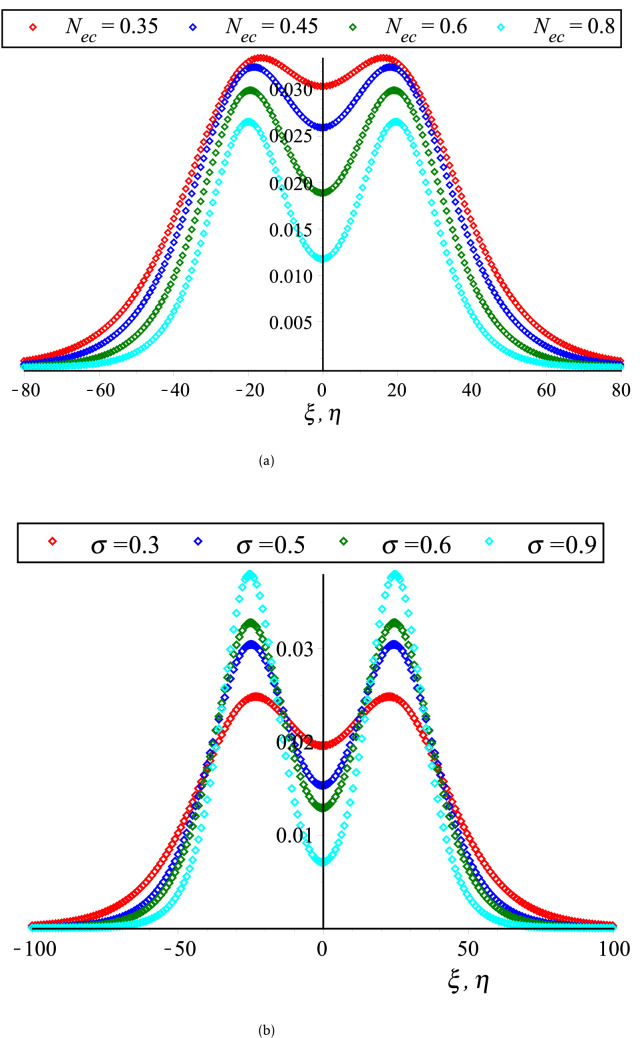
Collisional PA KdVSs $\left[ {\phi^{{({1})}} = \phi_{l}^{{({1})}} + \phi_{r}^{{({1})}} } \right]$ for different values of (**a**) *N*_*ec*_ (*σ* = 0.5) and (**b**) *σ* (*N*_*ec*_ = 0.5). The remaining dimensionless parameters are chosen as *N*_*hc*_ = 0.08 and *δ* = 1 with *U*_0_ = 0.01, *τ* = 2500, *r* = 0.1 and *q* = 3.

**Figure 5:**
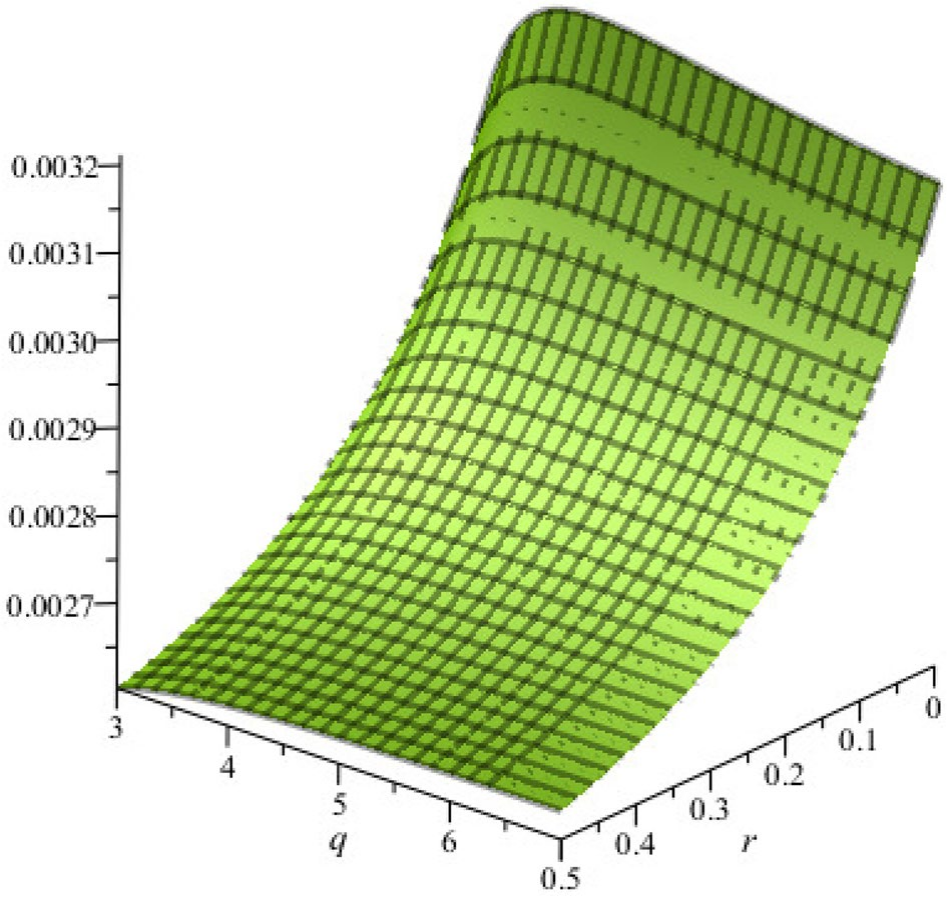
Influence of *q* and *r* on phase shift (Δ*Q*_0_) with *N*_*hc*_ = 0.08, *N*_*ec*_ = 0.5, $\epsilon$ = 0.1, *δ* = 0.5 and *σ* = 1.

In Fig. 2, the HOC between two equal amplitude PA KdVSs by varying $q$ and $r$ along with the constant parametric values of other parameters are displayed. This figure shows that both of compressive and rarefactive collisional PA KdVSs are supported in which the amplitudes and widths of PA KdVSs are decreasing (monotonically decreasing) with the increase of $r$ ($q$). Whereas, the influences of time ($\tau$), $\delta$, $N_{ec}$ and $\sigma$ on the HOC between two equal amplitude PA KdVSs are demonstrated in Figs. 3 and 4, respectively with the presence of Kappa and flat-topped distributions. These figures exhibit that the amplitudes and widths of interacting PA KdVSs are decreasing (monotonically increasing) with the increase of $N_{ec}$ and $\delta$ ($\sigma$). Besides, the interacting PA KdVSs becomes pulse like shaped with the changes of time. It is investigated from the above figures that the colliding PA KdVSs lose their energies monotonically with the increase of both superthermality on the tail of the velocity curve and the high energy particles on a broad shoulder of the velocity. But, the colliding PA KdVSs lose their energies without monotonically with the increase of the population of superthermal HEs and HPs, which is also in good agreement with the previous investigations^12,17^. In the physical point of view, the HEs and HPs are dynamically interacted with the MCPs with the losses of electron energy and MCPs density. As a result, the contribution of restoring force that provided by HPs and HEs pressure is decreased for the production of electrostatic resonances. However, the driving force (provided by M CPs inertia) is decreased because the growth of ion density is only interpreted as the depopulation of ions from the plasma system. It is also found from the above figures that the right to left propagating KdVSs is initially at $\xi = 0,\;\eta \to - \infty$, left to right propagating KdVSs is initially at $\eta = 0,\;\xi \to + \infty$ and after that they are asymptotically far away from each other. When $\tau \to \pm \infty$, the reverse situations are arisen, as it is expected. Later than competition of the processes of HOC between KdVSs, the stationary merged coherent structures are formed within $- \infty < t < + \infty$. Due to the collision of two counter propagating solitons along the trajectories, they are deviated far from their initial position. As a result, the time delays (phase shifts) are generated. To understand the influence of Kappa and flat-topped distributions on phase shift, the variation of phase shift with regards to $q$ and $r$ by considering the remaining parameters constant is displayed in Fig. 5. This figure shows that the phase shift is remarkably increased with the increase of $r$, while slightly increased up to $q \to 7$ and then almost remain unchanged with the increase of $q$. It is also found that the maximum growth rate are occurred with the absence of flat-topped parameter, that is $r = 0$. This means that the maximum growth of phase shift is produced with the presence of only super-thermal HEs and HPs.

**Figure 6:**
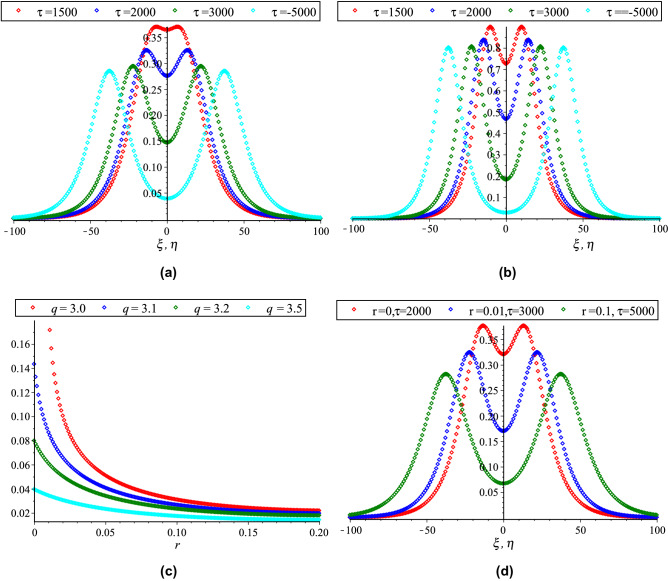
Collisional PA mKdVSs $\left[ {\phi^{{({1})}} = \phi_{l}^{{({1})}} + \phi_{r}^{{({1})}} } \right]$ with different values of *τ* around (**a**) *K*_*v*_ = 0.006,538,446,158 (*N*_*hc*_ = 0.08) and (**b**) *K*_*v*_ = 0.006,538,446,158 (*N*_*hc*_ = 0.1) with *N*_*ec*_ = 0.5, *δ* = 0.5, *σ* = 1, *q* = 3, *r* = 0 and *U*_0_ = 0.0075; (**c**) their phase shift Δ*Q*_0_ due to the collision between two PA mKdVSs with *N*_*hc*_ = 0.08, *N*_*ec*_ = 0.5, *δ* = 0.5, *σ* = 1, *q* = 3, *r* = 0, *ε* = 0.1 and *U*_0_ = 0.0075; and (**d**) effect of *r* (*q* = 3.5) on collisional PA mKdVSs with *N*_*hc*_ = 0.08, *N*_*ec*_ = 0.5, *δ* = 0.5, *σ* = 1, and *U*_0_ = 0.0075.

**Figure 7:**
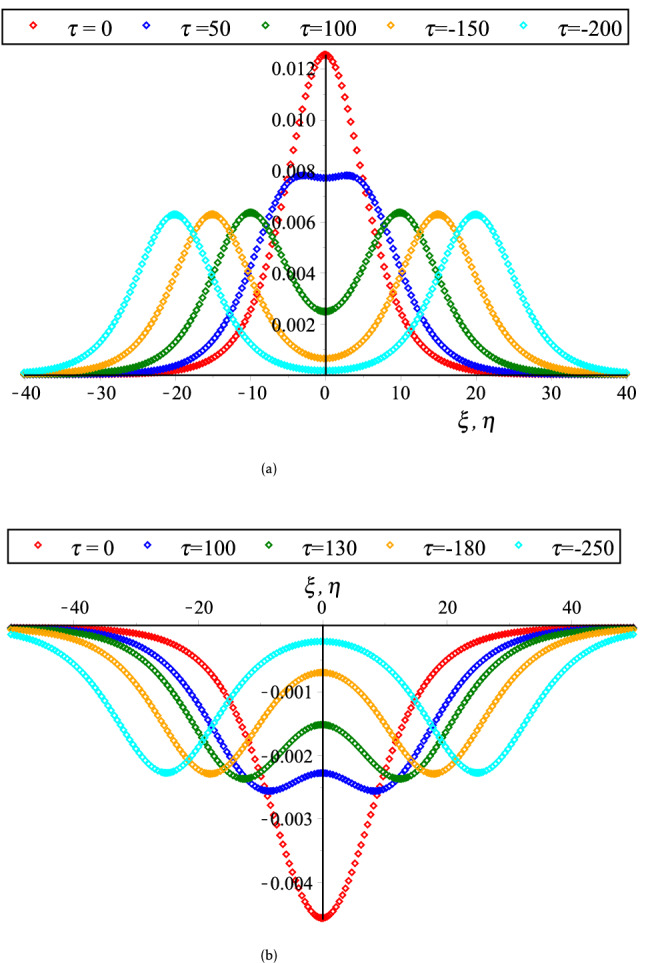
Electrostatic potential $\left[ {\phi_{l} = \phi_{l}^{(1)} (\xi ,\tau ) + \phi_{r}^{(1)} (\eta ,\tau )} \right]$ structures with different values of time due to head-on collision between GSs for (**a**) *N*_*hc*_ = 0.1 > *K*_*v*_ and (**b**) *N*_*hc*_ = 0.001 < *K*_*v*_ with *r* = 0, *q* = 3.5, *N*_*ec*_ = 0.4, *σ* = 3.5 and *δ* = 0.9.

**Figure 8:**
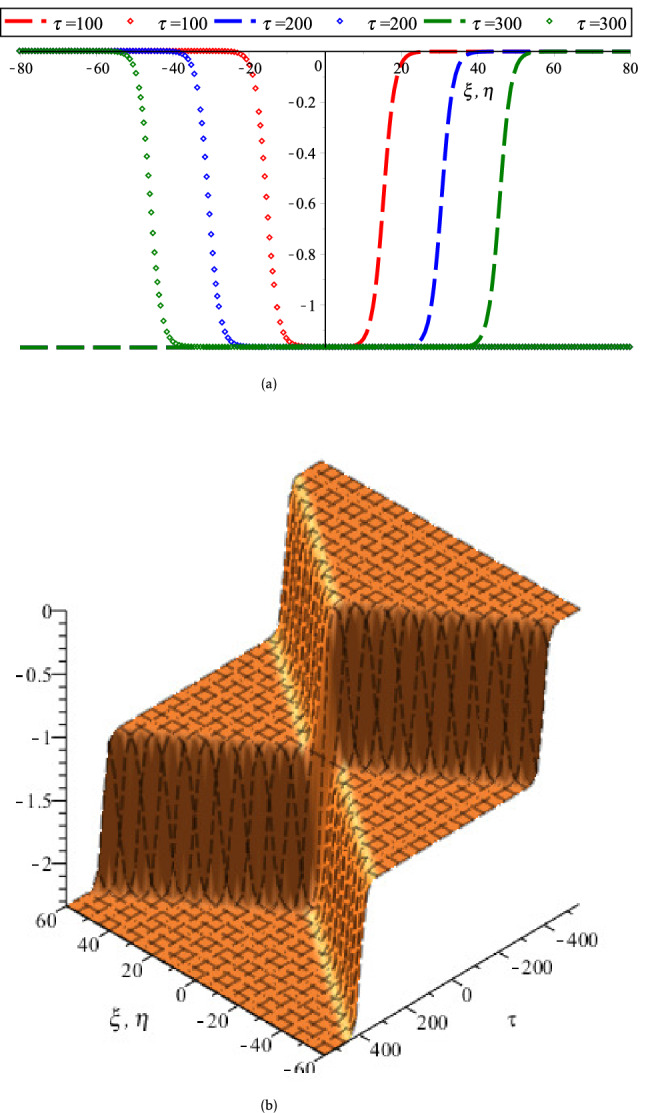
(**a**) Head-on collision between two equal amplitude DLs described by $\phi_{l}^{(1)} (\xi ,\tau )$ [Dashed dotted curves] and $\phi_{r}^{(1)} (\eta ,\tau )$ [point curves] for different values of time and (**b**) 3D collisional shock wave structures of electrostatic potential $\phi_{l} = \phi_{l}^{(1)} (\xi ,\tau ) + \phi_{r}^{(1)} (\eta ,\tau )$ for *N*_*hc*_ = 0.003 < *K*_*v*_ with *r* = 0, *q* = 3.5, *N*_*ec*_ = 0.4, *σ* = 3.5 and *δ* = 0.9.

**Figure 9:**
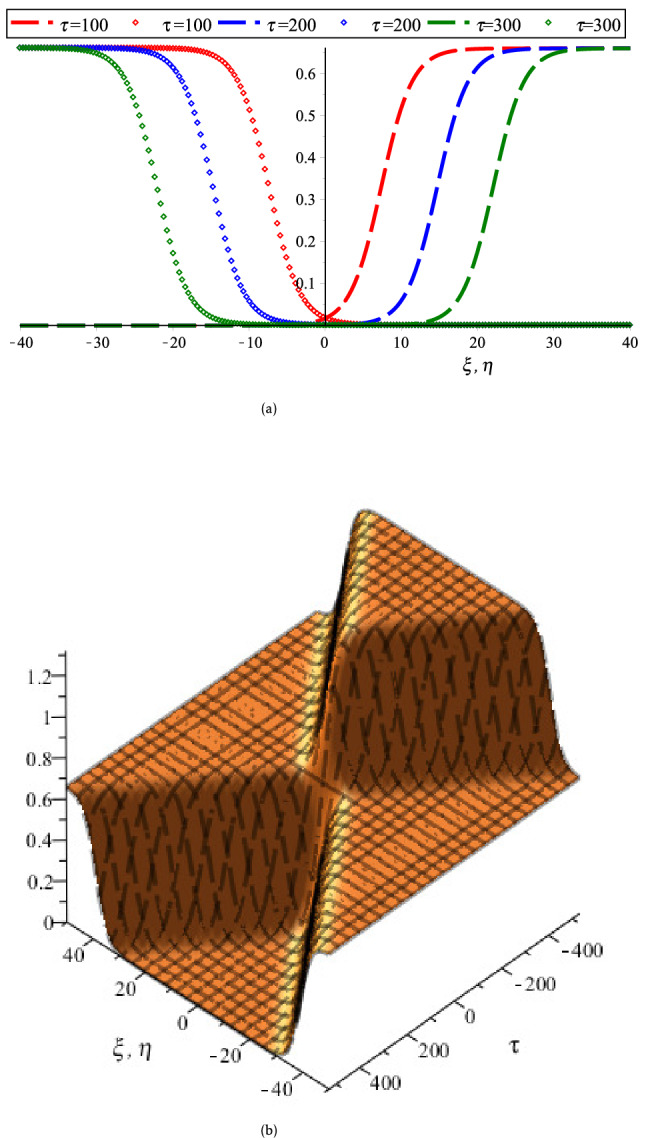
(**a**) Head-on collision between two equal amplitude DLs described by $\phi_{l}^{(1)} (\xi ,\tau )$ [Dashed dotted curves] and $\phi_{r}^{(1)} (\eta ,\tau )$ [point curves] for different values of time and (**b**) 3D collisional shock wave structures of electrostatic potential $\left[ {\phi_{l} = \phi_{l}^{(1)} (\xi ,\tau ) + \phi_{r}^{(1)} (\eta ,\tau )} \right]$ for *N*_*hc*_ = 0.1 > *K*_*v*_ with *r* = 0, *q* = 3, *N*_*ec*_ = 0.4, *σ* = 1 and *δ* = 0.5.

On the other hand, the coupled KdV equations are failed to address the collisional PA wave phenomena based on case (2). Because, the interacting KdVSs amplitude and their corresponding phase shifts are approaching to infinity at $K_{v}$ for any related parameters. To overcome such complicatedness, the coupled mKdV equations involving of higher order nonlinearity are derived based on case (2). By the useful solutions of such equations, the HOC between two-equal amplitude PA mKdVSs and their corresponding phase shifts around $K_{v}$’s with the presence of Kappa and flat-topped distributions are displayed in Fig. 6. These figures show that the interacting mKdVSs modify their polarities in the neighborhood of $K_{v}$ because such interacting solitons take up maximum energy with the change of maximum phase shifts. In addition, Fig. 6d is shown that the colliding solitons described by the coupled mKdV equations gain much more energies with the presence of only superthermal HEs and HPs, like the colliding solitons described by the coupled KdV equations. But, the amplitude of colliding mKdVSs and their corresponding phase shifts are comparatively higher rather than the colliding KdVSs and their corresponding phase shifts, which is in good agreement with the experimental and numerical investigations^42,50^. It is also observed that the interaction of mKdVSs takes lay only around $K_{v}$ which yields only the compressive two-counter propagating solitons with their shifting phases. However, the mKdV equations are failed to examine the nature of interacting wave phenomena at $K_{v}$ and any value less than $K_{v}$ because the nonlinear coefficient of mKdV equations are becomes complex at these points. In these situations, one needs to derive another coupled NLEEs to overcome such difficulty. Thus, the coupled Gardner equations are first time derived to study the nature of resonance wave phenomena not only around either less or greater than $K_{v}$ but also at $K_{v}$. It is observed that the coupled Gardner equations are divulged both of collisional PA solitons and DLs. Figure 7a and b show the electrostatic resonance potential due to HOC between two equal amplitude PA GSs propagating towards each other for $N_{hc} = 0.1 > K_{v}$ and $N_{hc} = 0.001 < K_{v}$, respectively. Whereas, Figs. 8 and 9 show the interaction of left to right ($\phi_{l}^{\left( 1 \right)} \left( {\xi , \tau } \right)$) and right to left ($\phi_{r}^{\left( 1 \right)} \left( {\eta , \tau } \right)$) propagating DLs and the electrostatic resonance potential due to HOC between two equal amplitude PA DLs for $N_{hc} = 0.1 > K_{v}$ and $N_{hc} = 0.001 < K_{v}$, respectively. These figures clearly indicate that both of compressive and rarefactive collitional PA GSs and DLs are produced in the considered plasmas. It is obviously observed that the right to left propagating PA GSs and DLs is initially at $\xi = 0,\;\eta \to - \infty$, left to right propagating PA GSs and DLs is initially at $\eta = 0,\;\xi \to + \infty$ and after that they are asymptotically far away from each other. When $\tau \to \pm \infty$, the reverse situations are arisen, as it is expected. Later than competition of the processes of HOC between PA GSs and DLs, the stationary merged coherent structures are formed within $- \infty < t < + \infty$. Figures 7, 8, and 9 also indicate that the maximum amplitudes of the colliding GSs and DLs are occurred not only around the critical but also at the critical number density ratios with the presence of Kappa and flat-topped distributions. It is predicted from the above discussion that the coupled Gardner equations are very useful rather that mKdV equations for describing the nature of both collisional PA solitons and DLs not only around the critical values but also at the critical values. It would be concluded from the above discussions that the theoretical outcomes might be very useful in understanding the nature of nonlinear propagation PA resonance solitons and PA resonance DLs in many SAEs, especially, in auroral acceleration regions, cosmic rays, solar wind, pulsar magmetosphere, and so on and in laboratory plasmas.

## Summary

In this article, the collisonal PA solitons and DLs have been investigated by formulating the coupled KdVs, mKdVs and Gardner equations in an unmagnetized plasma having IMPIs, MCPs, and Kappa with flat-topped distributed HPs and HEs. Note that the coupled Gardner equations have been derived for the first time to report not only the interaction of PA solitons but also PA DLs. The effects of plasma parameters on HOC between two-counter propagating solitons and DLs having equal amplitudes have been investigated. The outcomes show that the plasma parameters are notably modified the electrostatic potential resonances and their phase shift with the presence Kappa and flat-topped distribution in which the solitons energies remarkably minimized with the presence of the high energy particles on a broad shoulder of the velocity curve but gains much more energies with the presence of only HEs and HPs. It is also observed that the coupled KdV and Gardner equations are divulged both of compressive and rarefactive collitional solitons, whereas but only compressive collitional solitons with some limitations are supported by the coupled mKdV equations. In addition, the coupled Gardner equations are supported the PA collisonal DLs. It would be provided that the coupled Gardner equations must be very useful rather than mKdV equations for analyzing resonance wave dynamics in the plasmas.

## Data Availability

Data sharing is not applicable to this article as no new data were created or analyzed in this study.
