# Author Correction: Collisional positron acoustic soliton and double layer in an unmagnetized plasma having multi-species

**DOI:** 10.1038/s41598-022-21628-z

**Published:** 2022-10-20

**Authors:** Shahrina Akter, M. G. Hafez

**Affiliations:** 1 Department of Mathematics, Chittagong University of Engineering and Technology, Chattogram, 4349 Bangladesh; 2 Department of Mathematics, Premier University, Chattogram, Bangladesh

Correction to: *Scientific Reports* 10.1038/s41598-022-10236-6, published online 19 April 2022

The original version of this Article contained errors in the Mathematical analysis section, under the subheading ‘Coupled mKdV equations and its stationary solutions’. The width of mKdV solitons was incorrect.

$$ \phi_{1w} = \phi_{1a} \sqrt {\left( {M_{1} /6} \right) } $$

now reads:

$$ \phi_{1w} = \sqrt {\left( {M_{2} /U_{0} } \right)} $$

As a result, an updated version of Figure 6 has been supplied with changes in panels A, B, C and D. The original Figure 6 and accompanying legend appear below.

**Figure 6:**
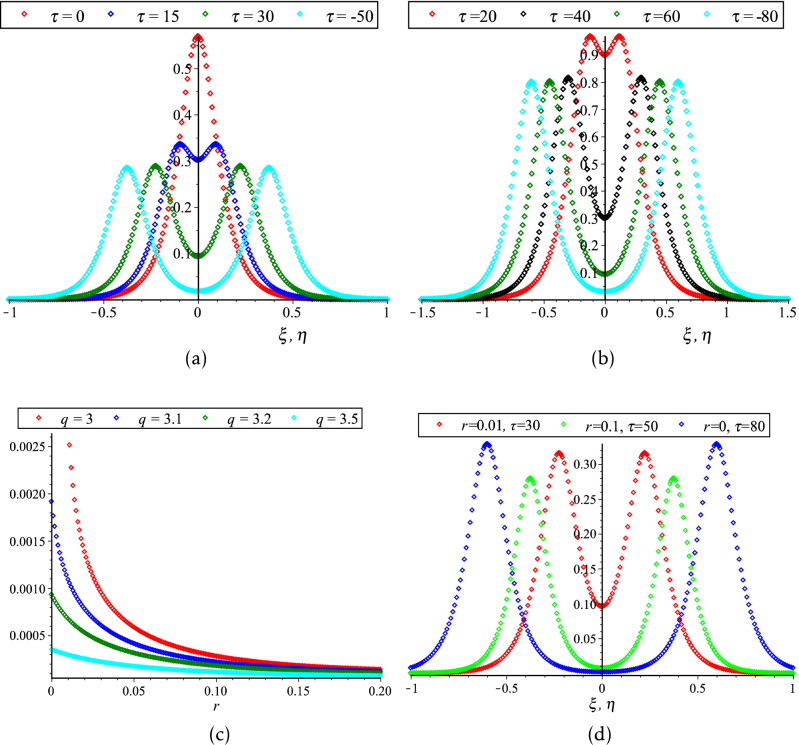
Collisional PA mKdVSs $\left[ {\phi^{\left( 1 \right)} = \phi_{l}^{\left( 1 \right)} + \phi_{r}^{\left( 1 \right)} } \right]$ with different values of τ around (**a**) *K*_*v*_ = 0.006,538,446,158 (*N*_*hc*_ = 0.08) and (**b**) *K*_*v*_ = 0.006,538,446,158 (*N*_*hc*_ = 0.1) with *N*_*ec*_ = 0.5, *δ* = 0.5, *σ* = 1, *q* = 3, *r* = 0 and *U*_0_ = 0.0075; (**c**) their phase shift Δ*Q*_0_ due to the collision between two PA mKdVSs with *N*_*hc*_ = 0.08, *N*_*ec*_ = 0.5, *δ* = 0.5, *σ* = 1, *σ* = 1,* q* = 3,* r* = 0 and *U*_0_ = 0.0075; and (**d**) effect of *r* (*q* = 3.5) on collisional PA mKdVSs with *N*_*hc*_ = 0.08, *N*_*ec*_ = 0.5,* δ* = 0.5, *σ* = 1, and *U*_0_ = 0.0075.

The original article has been corrected.

